# Management of Complex Gallstone Disease With Bouveret’s and Mirizzi Syndrome: A Case Report With Literature Review

**DOI:** 10.7759/cureus.101953

**Published:** 2026-01-20

**Authors:** Rami Ayoub, Omaymah Al-shweiki, Vandana Basappa Giriradder, Ali Yasen Mohamedahmed, Malik Kayal, Lydia Renardson, Konstantinos Baronos, Jawad Ahmad

**Affiliations:** 1 Hepatobiliary Surgery, Al-Balqa Applied University, Al-Balqa, JOR; 2 Hepatobiliary Surgery, University Hospitals Coventry and Warwickshire, Coventry, GBR; 3 Endocrinology, University Hospital Coventry and Warwickshire, Coventry, GBR; 4 General Surgery, University Hospitals of Derby and Burton National Health Service (NHS) Trust, Burton on Trent, GBR; 5 General Surgery, University Hospitals Coventry and Warwickshire, Coventry, GBR; 6 Hepatopancreatobiliary Surgery, University Hospitals of Coventry and Warwickshire, Coventry, GBR; 7 General Surgery, University Hospitals of Leicester National Health Service (NHS) Trust, Leicester, GBR

**Keywords:** bouveret's syndrome, gallstone disease (gsd), general surgery and hpb surgery, hpb surgery, mirizzi's syndrome

## Abstract

Gallstone disease affects up to 20% of adults, but rare complications include Bouveret’s syndrome, occurring in approximately 0.05% of patients with gallstones, and Mirizzi syndrome, with Type Va involving concurrent biliary and enteric fistulae. We report the case of a 64-year-old man with concurrent Bouveret’s syndrome and Csendes Type Va Mirizzi syndrome associated with a cholecystoduodenal fistula. An initial laparoscopic cholecystectomy was abandoned due to concern for malignancy. During the same admission, the patient developed gastric outlet obstruction, and imaging confirmed Bouveret’s syndrome caused by a large ectopic gallstone. This was managed with robotic-assisted gastrotomy and stone extraction as a damage-control procedure, with definitive biliary surgery deferred because of severe inflammation and unclear anatomy. Subsequent imaging demonstrated a residual Hartmann’s pouch stone with Type Va Mirizzi syndrome and a persistent cholecystoduodenal fistula. The patient underwent a robotic subtotal cholecystectomy with intraoperative indocyanine green assessment and endoscopic evaluation. His postoperative course was complicated by peritonitis, bilious and enteric leakage, and intra-abdominal collections, requiring laparoscopic washout, radiological drainage, total parenteral nutrition, and endoscopic retrograde cholangiopancreatography with placement of a covered metal stent for a cystic duct stump leak. The patient recovered with multidisciplinary management and remained well at follow-up. This case illustrates the complexity of managing dual fistula-related gallstone disease and highlights the importance of staged decision-making, detailed imaging, and combined surgical and endoscopic approaches.

## Introduction

Gallstone disease (cholelithiasis), a common gastrointestinal condition with a global adult prevalence up to 20% [1], often necessitates cholecystectomy in symptomatic cases. Although the majority of cases are asymptomatic, complications are well established and include acute cholecystitis, cholangitis, and pancreatitis. It is very rare for gallstone disease to present as Bouveret’s syndrome, a phenomenon characterized by gastric outlet obstruction secondary to migration of gallstones through a cholecystoenteric fistula, with an absolute incidence 0.05% of gallstone patients less than one in 10,000 [2]. This and similar conditions account for less than 3% of gallstone-related intestinal obstructions, posing diagnostic and therapeutic challenges [3].

Mirizzi syndrome is another rare complication of chronic gallstone disease. The Csendes classification describes Type I as extrinsic compression of the common hepatic duct, while Types II-IV represent progressive erosion and development of a cholecystobiliary fistula, with Type IV indicating an almost complete destruction of the bile duct wall. Type V refers to Mirizzi syndrome associated with a cholecystoenteric fistula; Va occurs without gallstone ileus, and Vb occurs with gallstone ileus. The simultaneous occurrence of Mirizzi syndrome and Bouveret’s syndrome is exceptionally rare, as it requires both a cholecystobiliary fistula and a cholecystoenteric fistula to form concurrently.

Bouveret's syndrome usually occurs in elderly patients with chronic cholecystitis and is characterized by symptoms of gastric outlet obstruction such as vomiting, abdominal pain, and weight loss [4]. Diagnosis is based on computed tomography (CT) and endoscopy, which help identify ectopic gallstones and define the level of obstruction [5]. For patients with larger or impacted stones, surgical intervention is often required, although endoscopic retrieval is attempted initially [6].

Recent evidence highlights the evolving role of advanced surgical and endoscopic techniques in managing complex cases like Bouveret’s syndrome [1,7,8].

We present a case of a 64-year-old man diagnosed with Bouveret’s syndrome, Csendes Type Va Mirizzi syndrome, and a D2 cholecystoduodenal fistula. Through this case and literature review, we highlight the diagnostic and management challenges associated with these rare complications.

## Case presentation

A 64-year-old man with a medical background of hypertension, ischemic heart disease, and obstructive sleep apnoea presented to the surgical assessment unit with acute on chronic right upper quadrant abdominal pain and intermittent nausea secondary to known cholelithiasis previously confirmed on abdominal ultrasound scan. He was scheduled for an emergency laparoscopic cholecystectomy. Intraoperatively, the gallbladder appeared to have local inflammation, unclear biliary anatomy and a suspicious mass. This raised concerns of malignancy and thus the procedure was abandoned with the plan to do further imaging tests.

**Figure 1:**
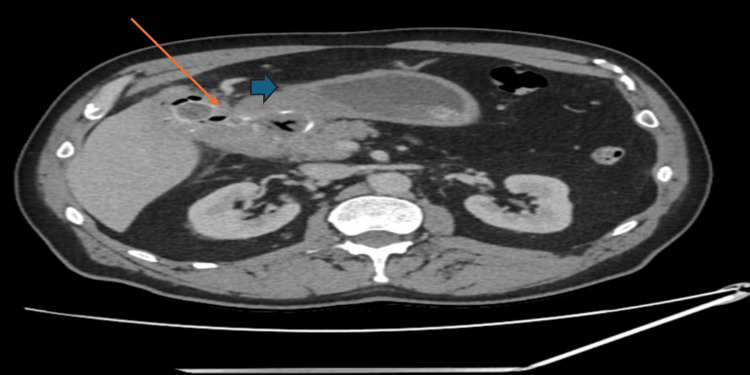
Computed tomography (CT) showing cholecytoduodenal fistula secondary to a chronic calculous CT scan demonstrating a 3 cm gallstone in the distended stomach (blue arrow) with pnuemobilia and collapse of the distal bowel loops. The thick-walled gallbladder contains multiple stones (orange arrow). These findings are consistent with Bouveret's syndrome.

During the same hospital admission, the patient developed persistent non-bilious vomiting, raising concerns for acute gastric outlet obstruction. Computed tomography (CT) demonstrated a large ectopic gallstone measuring approximately 3 cm within the stomach (Figure 1), consistent with Bouveret’s syndrome and no evidence of malignancy. He underwent robotic-assisted gastrotomy, during which three gallstones were removed - one from the stomach and two from the second part of the duodenum (D2). The patient had an uncomplicated early postoperative course and showed resolution of the obstructive symptoms.

**Figure 2:**
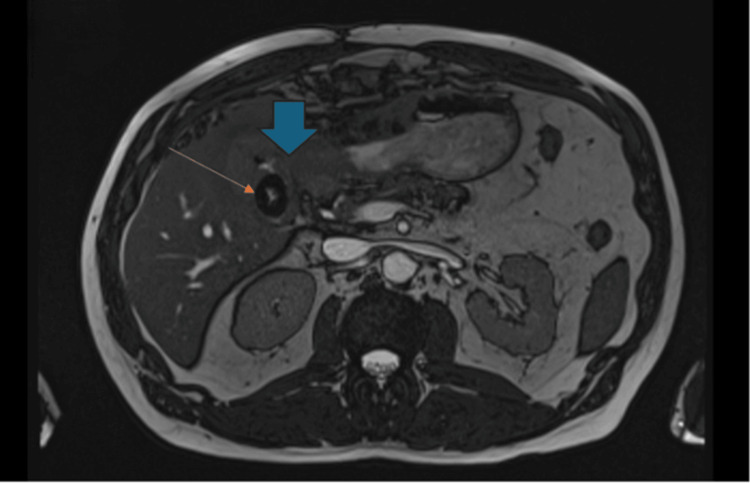
Post-gastrotomy magnetic resonance cholangiopancreatography (MRCP) Post-gastrotomy MRCP showing a large Hartmann's pouch stone (orange arrow) associated with Csendes Type Va Mirizzi syndrome. A cholecystoduodenal fistula opens into the second part of the duodenum D2 (blue arrow).

To further characterize the anatomy of the gallbladder and local structures, a magnetic resonance cholangiopancreatography (MRCP) was performed, which showed a large residual stone in Hartmann’s pouch with Type Va Mirizzi syndrome and a D2 cholecystoduodenal fistula (Figure 2). The patient then underwent robotic subtotal cholecystectomy and the infundibular gallbladder remnant was closed using an endoloop as a damage-control measure to avoid narrowing of the common bile duct, and indocyanine green fluorescence imaging (ICG) showed no bile leak. Intraoperative oesophago-gastro-duodenoscopy (OGD) confirmed a 5-8 mm fistula opening. The primary repair of the fistula was not undertaken due to friable tissue and concern for duodenal leak, and a damage-control strategy with external drainage and close postoperative monitoring was adopted.

In the following two days, the patient developed generalized peritonitis. Emergency laparoscopy revealed bile and undigested food within the subhepatic space, including loculated food debris around the porta hepatis. The gastrotomy staple line was intact, and no discrete duodenal defect amenable to direct repair was identified. The detection of food particles within the porta hepatis strongly suggests that the leak originated from the duodenal aspect of the cholecystoduodenal fistula rather than the gallbladder remnant or biliary system. Enteric leakage following subtotal cholecystectomy in Mirizzi syndrome is a recognized risk when a cholecystoenteric fistula is present, particularly if the duodenal component is not primarily repaired. The contamination was therefore attributed to backflow through the pre-existing cholecystoduodenal fistula and was managed with thorough lavage, placement of a wide-bore surgical drain in the subhepatic space, and a planned endoscopic diversion of bile. Daily drain output reached 200-400 ml. He was managed with oral parenteral nutrition (TPN) for seven days and broad-spectrum antibiotics. Laboratory investigations and serial inflammatory markers from admission through deterioration are summarized in Table 1.

**Table 1:** Serial Laboratory Findings (July 19 to August 14, 2024) Reference ranges: White blood cells (WBC): 4-11×10⁹/L; C-reactive protein (CRP) <10 mg/L; total bilirubin <21 µmol/L; amylase 30-118 IU/L; alanine aminotransferase (ALT) 10-49 U/L; alkaline phosphatase (ALP) 30-130 U/L. Trend summary: Post-operative CRP peaked (~160 mg/L), declined and then showed a secondary rise before settling. Liver enzymes and ALP gradually improved toward discharge. POD: Post-operative day.

Date	WCC (×10⁹/L)	CRP (mg/L)	Bilirubin (µmol/L)	Amylase (U/L)	ALT (U/L)	ALP (U/L)	Albumin (g/L)	INR	Clinical milestone
Reference range and units	4.0-11.0	0-10	1.7-20.5	30-110	7-55	30-130	35-55	0.8-1.2	
July 19 POD 0	7.8	18	8	55	42	110	38	1.0	Robotic gastrotomy
July 23 POD 4	9.6	160	10	58	90	240	35	1.1	Peritonitis, washout
July 27 POD 8	10.8	110	12	56	85	300	33	1.1	Bilious/enteric leak (200–400 ml/day)
July 31 POD 12	9.2	140	9	57	70	260	34	1.0	Collections enlarging
August 4 POD 16	8.4	95	8	56	60	220	36	1.0	Radiological drainage
August 8 POD 20	8.1	60	9	55	52	320	37	1.1	Endoscopic retrograde cholangiopancreatography with self-expanding metal stent placement
August 14 POD 26	8.8	68	7	56	62	223	39	1.0	Discharge

**Figure 3:**
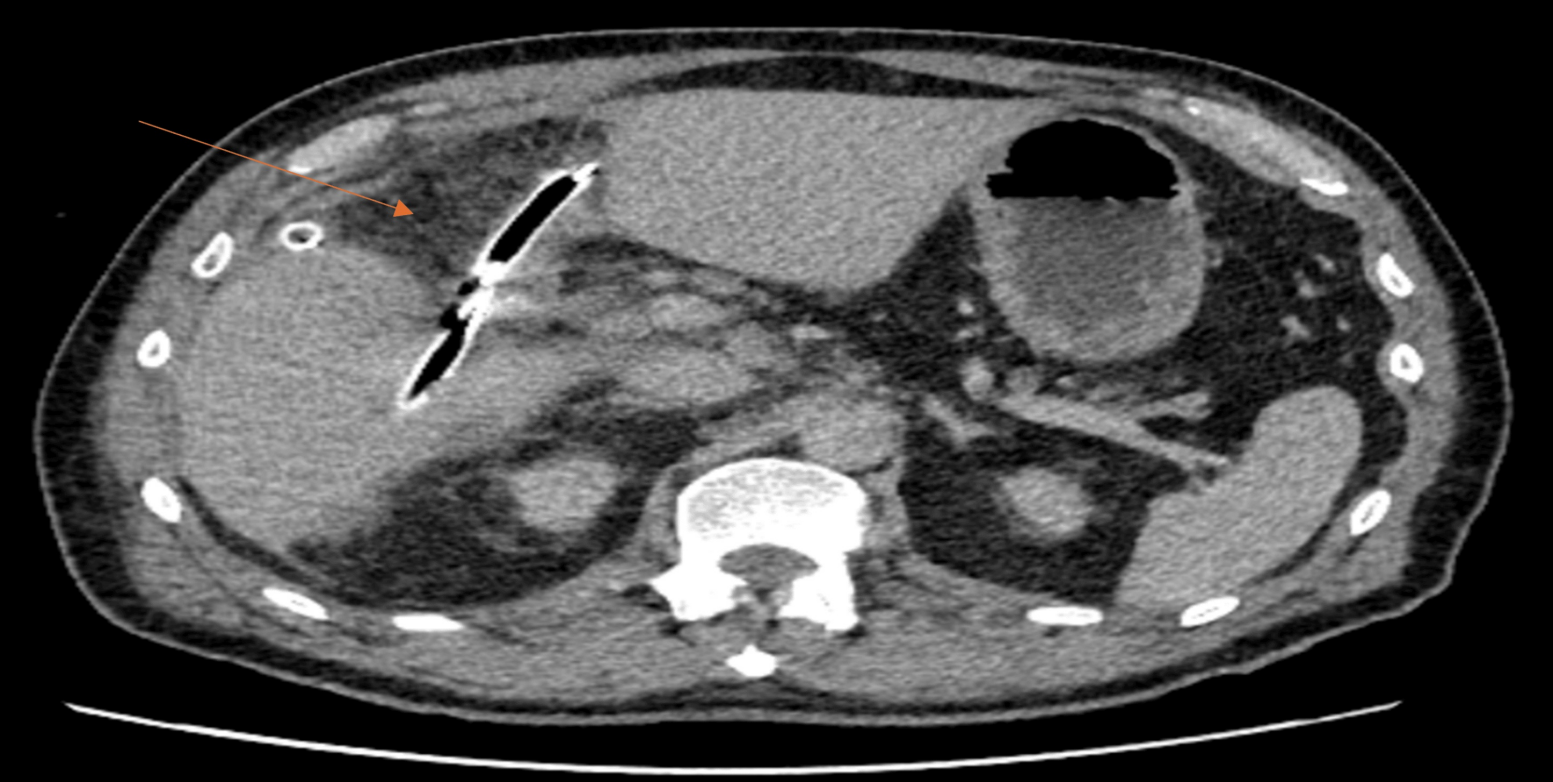
CT showing subscapular collection CT abdomen demonstrating enlarging subcapsular collections in segments VI-VII with intralesional gas, suggestive of secondary infection.

**Figure 4:**
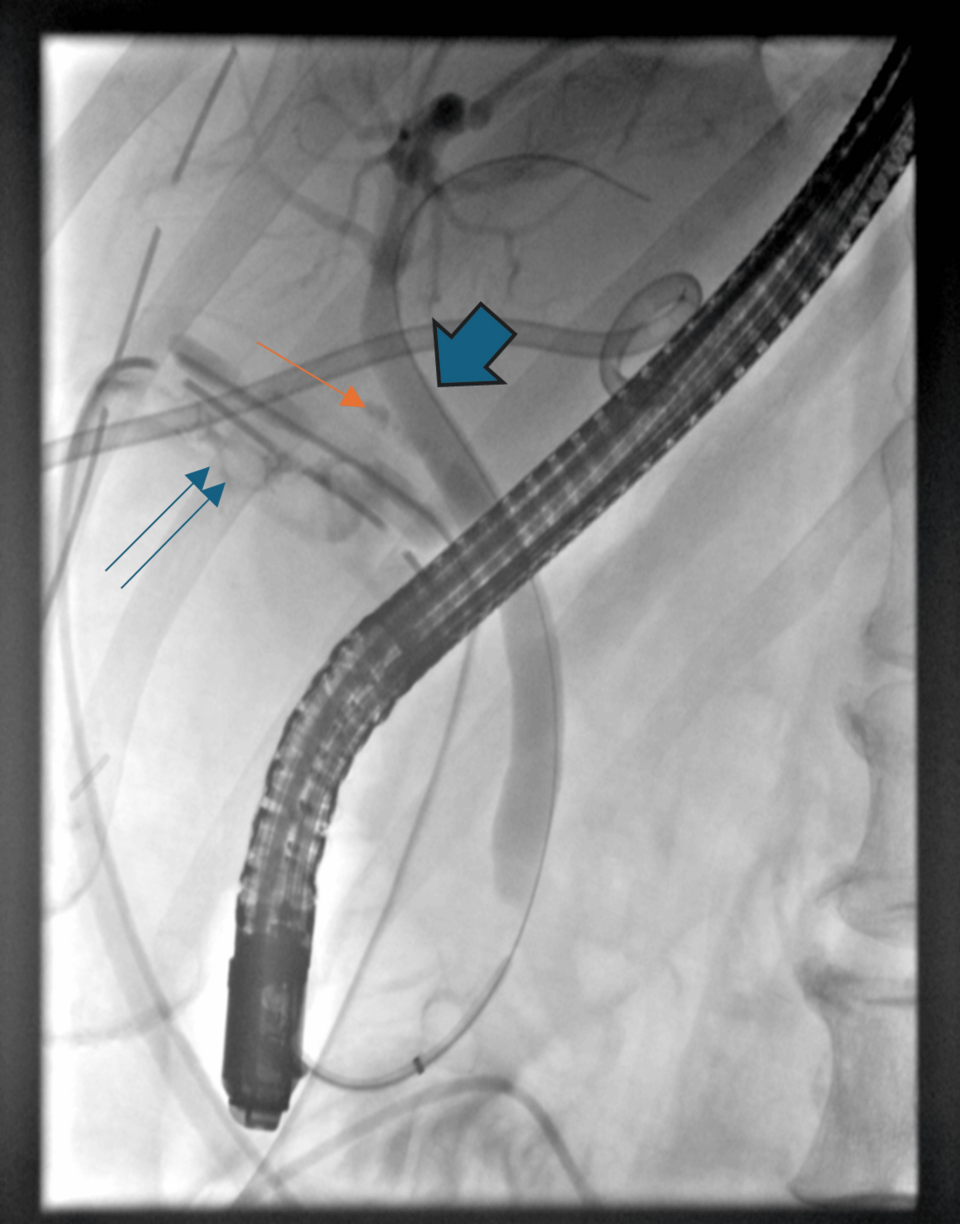
Endoscopic retrograde cholangiopancreatography (ERCP): Bile leak ERCP demonstrating a bile leak originating from the cystic duct stump leak (double blue arrow).The cystic duct stump is opacified (single arrow). The common bile duct is shown by the large blue arrow.

Subsequent MRCP and CT showed enlarging subcapsular hepatic collections with intralesional gas (Figure 3). Radiological drainage was performed. Culture of the drained collection did not grow any organisms, and sepsis was controlled with drainage and antibiotics. Endoscopic retrograde cholangiopancreatography (ERCP) demonstrated a cystic duct stump leak, and a 10 mm×6 cm fully covered self-expandable metal stent (SEMS) was placed with sphincterotomy (Figure 4). Drain output gradually decreased, serial imaging demonstrated resolution of intra-abdominal collections and the drains were sequentially removed.

The patient was discharged on postoperative day 27, tolerating a normal diet and mobilizing independently. At three-month follow-up, MRCP demonstrated normal biliary anatomy, and the biliary stent was removed endoscopically. The patient returned to baseline function with no recurrent biliary symptoms.

## Discussion

Bouveret's syndrome was first described by Leon Bouveret in 1896 as "obstruction duodénale par calcul." It represents gastric outlet obstruction caused by a gallstone passing through a cholecystoenteric fistula. It accounts for less than 3% of gallstone ileus cases and remains diagnostically challenging [1-3]. Globally, around 550-600 cases have been reported to date [9,10].

Mirizzi syndrome is a rare complication of chronic gallstone disease involving extrinsic compression or erosion of the common hepatic duct by an impacted gallstone. In this case, the presence of a Type IV Mirizzi syndrome (complete cholecystobiliary fistula) concurrently with Bouveret's syndrome represents an exceptional combination. This dual pathology significantly influenced operative planning, postoperative complications, and the requirement for multimodal management including surgery, interventional radiology, and ERCP-based therapy.

Clinical presentation is nonspecific-vomiting, abdominal pain, weight loss-and may mimic malignancy or ulcer disease [1,4-6]. CT may show Rigler's triad (ectopic gallstone, pneumobilia, obstruction), present in 20%-35% of cases [1,8]; in our case, CT showed pneumobilia and a gastric stone.

Bouveret's syndrome is diagnosed via CT and endoscopy [1,2,4,5]. Endoscopy allows visualization and occasionally treatment, though success rates for extraction are only ~10% [1,5,10], necessitating surgery in most cases [1,2-4,8]. A summary of surgical management of Bouveret's syndrome is presented in Table 2.

**Table 2:** Summary of Studies on Bouveret’s Syndrome ERCP: Endoscopic retrograde cholangiopancreatography; SEMS: self-expandable metal stent

Year	Author	Age / Sex	Stone Size	Fistula Type	Treatment Performed	Complications	LOS	Outcome	Mirizzi Syndrome
2024	Chatterjee and De [1]	68 F	3-4 cm	Cholecystoduodenal	Failed endoscopy → Gastrotomy	None	10 days	Recovered	No
2024	Ranjan et al. [2]	72 M	3 cm	Cholecystogastric	Failed endoscopy → Laparotomy	Wound infection	14 days	Recovered	No
2023	Malik et al. [3]	80 F	Not reported	Cholecystogastric	Open gastrotomy	None	8 days	Recovered	No
2021	Smith et al. [4]	73 M	4 cm	Cholecystoduodenal	Laparoscopic enterolithotomy	None	7 days	Recovered	No
2021	Jin and Naidu [5]	90 M	3.5 cm	Cholecystoduodenal	Endoscopic lithotripsy	None	5 days	Recovered	No
2020	Osman et al. [6]	78 F	4 cm	Cholecystogastric	Failed endoscopy → Open surgery	None	9 days	Recovered	No
2020	Al-Saadi et al. [7]	79 F	Not reported	Cholecystogastric	Gastrotomy	None	Not reported	Recovered	No
2020	Singh et al. [8]	82 F	3 cm	Cholecystoduodenal	Failed endoscopy → Surgery	None	12 days	Recovered	No
2012	Lee et al. [9]	75 F	Not reported	Cholecystoduodenal	Open enterolithotomy	None	10 days	Recovered	No
2009	Doycheva et al. [10]	85 F	Not reported	Cholecystogastric	Endoscopic lithotripsy	None	6 days	Recovered	No
2024 (Current sase)	Ayoub et al.	64 M	3 cm	Cholecystoduodenal + Mirizzi Type Va	Robotic gastrotomy+Subtotal cholecystectomy +ERCP (covered SEMS)	Peritonitis + Cystic duct stump leak + Infected collections	27 days	Recovered	Yes

Mirizzi syndrome refers to external compression or erosion of the bile duct by an impacted gallstone. Csendes Type Va, as in our patient, involves near-complete destruction of the bile duct wall and formation of a cholecystobiliary fistula. The coexistence of Mirizzi and Bouveret's syndromes is exceptionally rare because it requires simultaneous development of two fistulas - one biliary and one enteric.

Management typically follows a stepwise approach: attempted endoscopic removal, followed by surgery when unsuccessful [1,2,5,10]. Larger or impacted stones require gastrotomy or enterolithotomy [1,2,4,8]. Robotic assistance may help in difficult cases with intense inflammation; in our case, dense adhesions and distorted anatomy made the robotic approach suitable.

Despite early intervention, our patient developed significant postoperative morbidity, including peritonitis, infected collections, and a cystic duct stump leak requiring laparoscopic washout, radiological drainage, ERCP, and TPN. This reflects the severity of disease when dual fistulas are present.

The decision to use a robotic approach was based on the anticipated difficulty caused by dense inflammation and distorted anatomy. High-definition 3D vision and wristed instruments facilitated dissection around the hepatoduodenal ligament and allowed safe identification of key structures despite the presence of dual fistulas. Although robotic management of Bouveret's syndrome is rarely reported, in this case, it provided enhanced control during both gastrotomy and subsequent subtotal cholecystectomy.

Advances in imaging, endoscopic therapy, and minimally invasive surgery have improved outcomes [1,5,10], although morbidity remains high in complex cases [2,8]. Multidisciplinary care is essential.

## Conclusions

Bouveret’s syndrome is a rare but significant complication of gallstone disease, and its coexistence with Type Va Mirizzi syndrome is exceptionally uncommon. This case highlights the importance of thorough imaging, intraoperative endoscopy, and multidisciplinary management.

Despite careful planning and modern techniques such as robotic surgery and ICG assessment, our patient experienced major postoperative complications, including peritonitis and a cystic duct stump leak. Early recognition of dual fistulas, staged operative planning, and prompt management of postoperative complications are critical in achieving a successful recovery.
